# College and Hospital Notes

**Published:** 1906-05

**Authors:** 


### COLLEGE AND HOSPITAL NOTES.

The University of Buffalo is planning to hold a commencement of the four departments on June 1, 1906. The details are not definitely arranged at the writing of these lines, but we feel sure the commencement this year will be an important one, as it will serve to accentuate the work that has been in hand for some time past relating to the establishment of a college of arts. Every alumnus or alumna should take an interest in this matter, which should be manifested by a large attendance at the commencement ceremonies and the annual meeting of the alumni association.

---

The New York State Hospital for the care of crippled and deformed children, which is located at West Haverstraw, on the West Shore Railroad, has lately issued its fifth annual report, which is for the year ending September 30, 1905. It treated fifty-six patients during this period, one of whom was from Erie County. A friend of the hospital has contributed $500, to be used in transporting eligible, indigent patients living in the rural regions, to and from the hospital. This deserving charity is presided over by Bishop Potter, and Dr. Newton M. Shaffer is the surgeon-in-chief.

---

The College of Medicine of Syracuse University has a four years' course of study. Its facilities for instruction are unusually good. They consist of a main building erected in 1896, containing a library of several thousand volumes, study and lecture rooms, and laboratories large, light, convenient and well equipped with the apparatus required in the study of the fundamental medical sciences. Its facilities also include access to three large hospitals and three institutions for purposes of clinical instruction. It has a faculty of progressive, able teachers, who are doing splendid work in equipping a large number of undergraduates for the practise of medicine.

---

Oak Grove, a hospital for the treatment of nervous and mental diseases which was established at Flint, Mich, 1891, has issued an album of pictures and text descriptive of its methods, purpose, location, size and structure. It is a handsome specimen of the printer's and engraver's arts, filled with large and small halftones of exterior and interior views of the hospital, many of its pages being decorated with oak leaves. The hospital is beautifully located in the midst of an oak grove, and surrounded by a fine agricultural region. It is presided over by Dr. C. B. Burr, as medical director, who is one of the most accomplished alienists of the period.

The Hospital Aid Association of Lockport held a meeting and adopted a resolution asking the Common Council to proceed with the work of the new City Hospital. The association has raised $5,000, the city appropriates $5,000 and gives a site. The association further requested that it be given representation on committees appointed for plans, etc., relative to the new hospital.

---

Saint Leo's Hospital, at Greensboro, N. C., was opened by the Sisters of Charity, April 18, 1906. The Journal acknowledges an invitation to attend the ceremonies.

